# Insurance Coverage Mandates and the Adoption of Digital Breast Tomosynthesis

**DOI:** 10.1001/jamanetworkopen.2022.4208

**Published:** 2022-03-25

**Authors:** Ilana B. Richman, Jessica B. Long, Kelly A. Kyanko, Xiao Xu, Cary P. Gross, Susan H. Busch

**Affiliations:** 1 Section of General Internal Medicine, Department of Internal Medicine, Yale School of Medicine, New Haven, Connecticut; 2 Cancer Outcomes, Public Policy, and Effectiveness Research Center, Yale Cancer Center and Yale University School of Medicine, New Haven, Connecticut; 3 Department of Population Health, New York University School of Medicine, New York; 4 Department of Obstetrics, Gynecology and Reproductive Sciences, Yale School of Medicine, New Haven, Connecticut; 5 Department of Health and Policy Management, Yale School of Public Health, New Haven, Connecticut

## Abstract

**Importance:**

Digital breast tomosynthesis (DBT) is a breast cancer screening modality that has gained popularity in recent years. Although insurance coverage for DBT is not mandated under the Patient Protection and Affordable Care Act, several states have required coverage without cost sharing for private insurers.

**Objective:**

To evaluate the association between state-level insurance coverage mandates for DBT and changes in DBT use, price, and out-of-pocket payments.

**Design, Setting, and Participants:**

This cohort study used an event-study design with repeated cross-sectional observations of US states. Data were obtained from the Blue Cross Blue Shield Axis database for commercially insured women aged 40 to 64 years who underwent screening mammography between January 1, 2015, and June 30, 2019. Data were analyzed between January 14, 2021, and January 20, 2022.

**Interventions:**

Passage of state-level legislation requiring insurance coverage of DBT.

**Main Outcomes and Measures:**

Change in DBT use among women screened for breast cancer, overall DBT price, and out-of-pocket payments for DBT in states with mandates for coverage of DBT compared with states that did not pass legislation.

**Results:**

This study included 9 604 084 screening mammograms from 5 754 123 women (mean [SD] age, of 53 [6.7] years). During the study period, 15 states enacted DBT coverage mandates and 34 states did not. In states with coverage mandates, DBT use increased by 9.0 percentage points (95% CI, 1.8-16.3 percentage points; *P* = .02) 2 years after the mandate compared with states without coverage mandates. Coverage mandates were also associated with a net $38.7 (95% CI, $13.4-$63.9; *P* = .003) decrease in the mean price of DBT compared with no coverage mandates. There was no association between coverage mandates and out-of-pocket payments 2 years after mandate passage ($–2.1; 95% CI, $–5.3 to $1.0; *P* = .18).

**Conclusions and Relevance:**

In this cohort study, DBT coverage mandates were associated with an increase in DBT use but not with any change in out-of-pocket payments. The findings suggest that coverage mandates for DBT may have been associated with broader use but were not associated with changes in direct costs to patients.

## Key Points

**Question:**

Are state-level mandates that require insurance coverage of digital breast tomosynthesis (DBT) for breast cancer screening associated with changes in DBT use and price?

**Findings:**

In this cohort study of 9 604 084 screening mammograms from 5 754 123 women, insurance coverage mandates were associated with greater DBT use and a decrease in overall DBT price but not in out-of-pocket payments.

**Meaning:**

In this study, results suggest that mandates may be associated with DBT adoption through mechanisms other than reduction of financial liability for patients.

## Teaser

This cohort study assesses the association of state-level mandates requiring coverage of digital breast tomosynthesis (DBT) for breast cancer screening with DBT use, price, and out-of-pocket payments.

## Introduction

Over the past decade, breast cancer screening has undergone a substantial technological shift in the US in which digital breast tomosynthesis (DBT) has supplanted standard 2-dimensional (2D) mammography alone as the standard of care.^1,2^ Digital breast tomosynthesis uses multiple x-ray exposures to create a pseudo–3-dimensional image of the breast and is typically interpreted in conjunction with standard 2D imaging.^3^ Studies of DBT have shown that when used for breast cancer screening, DBT may be associated with reduced likelihood of a woman being called back for additional imaging because of an abnormality seen on the initial screening test.^4^ Digital breast tomosynthesis may also be more sensitive than 2D digital mammography for detecting breast cancer.^5^ However, important questions remain about whether DBT improves health outcomes compared with 2D mammography, and major guidelines, including those from the US Preventive Services Task Force and the American Cancer Society, make no specific recommendation for or against the use of DBT.^6,7^

The US Food and Drug Administration approved the first DBT system for breast cancer screening in 2011. Four years later, Medicare began covering DBT as an add-on charge when performed in conjunction with 2D mammography.^8,9^ Private insurers have not been required to cover DBT under the Patient Protection and Affordable Care Act because DBT has not received an A or B recommendation by the US Preventive Services Task Force.^6,10^ Absent a federal mandate, many private insurers did not immediately cover DBT, characterizing DBT as elective and citing the lack of long-term data.^11,12^ However, to date, 17 states have enacted laws that require private health insurers to cover DBT without cost sharing. Two additional states have enacted coverage legislation but with some restrictions (eg, coverage only for women with dense breasts or coverage with cost sharing).^13^

Insurance benefit mandates such as these have been widely used in other contexts as a policy tool to protect consumers from high out-of-pocket costs and to facilitate access to important health services. However, benefit mandates can have complex effects and have been criticized for contributing to higher insurance premiums (and thereby potentially decreasing insurance rates), reducing the flexibility of plan design, and increasing the price of the specific mandated services by reducing negotiating power.^14,15^

The goal of this study was to evaluate the association between state DBT coverage mandates and DBT use, prices, and out-of-pocket payments. We used longitudinal data from a large administrative database to characterize state-level changes in DBT use, overall price, and out-of-pocket payments among privately insured women in the US after passage of a coverage mandate. We hypothesized that DBT coverage mandates would be associated with increased DBT use by protecting patients from cost sharing but that it would have variable associations with DBT price.

## Methods

### Study Population and Data

This cohort study used data from the Blue Cross Blue Shield Axis database, a deidentified database of health insurance claims. This data set includes claims from a large population of members in all 50 states. The geographic diversity of the sample and its longitudinal structure make it well suited to evaluate policies that vary by state. Within this data set, we identified screening mammograms performed among women 40 to 64 years of age between January 1, 2015, and June 30, 2019, using a validated algorithm (eTable 1 in the Supplement).^6^ Data were analyzed between January 14, 2021, and January 20, 2022. We excluded women aged 65 years or older because the Blue Cross Blue Shield Axis database does not include Medicare or Medicare Advantage claims and older women included in Blue Cross Blue Shield data may be highly selected. We used patient-level data to describe characteristics of the women and mammograms included in the study. All other analyses were performed at the state level. This study was considered exempt from institutional review board review and the requirement for informed consent by the Yale Human Investigations Committee because it was not considered human participants research. We followed the Strengthening the Reporting of Observational Studies in Epidemiology (STROBE) guideline.

### Exposure Definition

The exposure in this study was passage of a legislative mandate requiring coverage of DBT (yes or no) during the study period (eTable 2 in the Supplement). All states included as mandate states in this analysis also eliminated cost sharing with the exception of Connecticut, which eliminated cost sharing 2 years after passage of a general coverage mandate. Cost sharing generally included patients’ out-of-pocket payments toward deductibles, coinsurance, or copayments. We excluded 1 state (Indiana), which passed a mandate that required coverage of DBT only for women with dense breasts because measurement of breast density was not included in our data. Nonmandate states included all states that had not passed a coverage mandate during the observed period (hereafter referred to as nonmandate states). Mammograms were assigned to a state based on the location of the billing service.

### Outcome Definitions

We evaluated DBT use, mean price of the screening test (DBT or 2D mammography alone), mean screening price among women screened overall, proportion of women screened with DBT who had out-of-pocket payments, and mean out-of-pocket payment among women screened with DBT. All outcomes were calculated on the state level at 6-month intervals between January 1, 2015, and June 30, 2019. Prices were inflation adjusted to 2019 dollars using the universal Consumer Price Index for urban households.^16^

We defined DBT use as the proportion of screening mammograms performed with DBT among all screening mammograms for a state in a 6-month period. Digital breast tomosynthesis is typically read and billed in conjunction with standard 2D imaging. Thus, we considered screening DBT to have been performed when there was a claim for DBT in conjunction with a claim for screening mammography, as defined by the algorithm (eTable 1 in the Supplement).

We defined the screening mammography price as the total allowed amount for the initial screening test (2D alone or with DBT) reported on a claim. The total allowed amount included facility fees, professional fees, and any out-of-pocket payments. We considered out-of-pocket payments to include any amount paid as part of a deductible, copayment, or coinsurance noted on a claim. We calculated the proportion of women with any out-of-pocket payment from among women screened with DBT in each state in each 6-month period. We calculated the mean out-of-pocket payment only among women screened with DBT because most women screened with 2D mammography already had no cost sharing, as mandated by the Patient Protection and Affordable Care Act.

### Statistical Analysis

We used standard descriptive statistics including means and proportions to describe the study population using individual-level data. To evaluate the association between state insurance coverage mandates and outcomes, we used an event-study design. An event-study design estimates changes in an outcome among states that passed a law compared with changes among states that had not passed a law at each 6-month interval after enactment of legislation. This specification allows for the effect of legislation to vary over time.

To implement this model, we fit a linear regression model with a series of binary covariates indicating time since passage of legislation in 6-month intervals using data aggregated at the state level. Indicators took on a value of 1 if the state had enacted legislation in the specified time interval (eg, 6 months or 1 year prior to the specified time interval) and 0 otherwise. Indicators were always 0 for states that never passed a mandate. We defined period 0 as the first full 6-month period for which legislation was in effect. For states that enacted legislation on January 1 or July 1, period 0 began on those dates. Period −0.5 included the period during which legislation was enacted and was used as the reference period (eFigure 1 in the Supplement).

Models included state fixed effects, which captured time-invariant differences among states, and year fixed effects to account for temporal changes present across states. We clustered SEs by state to account for correlation of repeated observations for a state. We also weighted models by the size of the screened population in each state for each period. All *P* values were 2-tailed, and we considered a *P* value of <.05 to indicate statistical significance.

In sensitivity analyses, we also adjusted our model of DBT price for 2D mammography price. We separately modeled the difference between DBT price and 2D mammography price. These models were intended to account for the fact that 2D mammography price may change over time independently of any policy change and may affect DBT price because DBT is billed as an add-on to 2D mammography. We also performed sensitivity analyses, adjusting for the proportion of women in the screened population with administrative services–only plans, which are typically not subject to state mandates.

As an alternative to the event-study design, we used a canonical 2 × 2 difference-in-differences design. We limited intervention states to the 4 states that enacted coverage mandates between January 1, 2016, and January 1, 2017. Control states included all nonmandate states during the study period, excluding Indiana. This simplified specification allowed us to model a single preperiod and postperiod with balanced event time (eMethods in the Supplement). We used Stata, versions 16.0 SE and 14.2 SE (StataCorp LLC) to conduct statistical analysis.

## Results

The study included 9 604 084 screening mammograms from 5 754 123 women performed between January 1, 2015, and June 30, 2019. During this period, 15 states passed DBT coverage mandates (eTable 2 and eFigure 1 in the Supplement) and 34 states did not enact a mandate. At the start of the study period, the mean (SD) age in the overall cohort was 53.0 (6.7) years, with women in mandate and nonmandate states having similar ages (mean [SD] age in both groups, 53.0 [6.7] years) (Table). Among women in mandate states, 42% lived in the Northeast and 7% lived in the Midwest. Among women in nonmandate states, 12% lived in the Northeast, and 29% lived in the Midwest (Table).

**Table.** Patient Characteristics, DBT Use, and DBT Price by Mandate Status at Baseline^a^

Characteristic	Mandate states	Nonmandate states
Screening mammograms, No.	333 929	615 511
Age, mean (SD), y	53.0 (6.7)	53.0 (6.7)
Metropolitan vs rural areas, No. (%)	284 937 (85)	518 129 (84)
Census region, No. (%)		
Midwest	23 376 (7)	180 942 (29)
Northeast	140 009 (42)	74 047 (12)
South	134 467 (40)	290 911 (47)
West	35 717 (11)	69 611 (11)
Price, mean (SD), $		
DBT	311.36 (131.80)	347.05 (149.43)
2D mammography	265.91 (108.24)	252.23 (109.62)
Any mammography	273.11 (113.52)	262.25 (118.13)
DBT use		
No. (%)	52 899 (16)	65 014 (11)
Any out-of-pocket payment, No. (%)	3500 (7)	4776 (7)
Out-of-pocket payment, mean (SD), $	5.13 (29.93)	5.17 (29.90)
Cost of any out-of-pocket payment, mean (SD), $	77.47 (89.06)	70.42 (82.34)

Abbreviations: 2D, 2-dimensional; DBT, digital breast tomosynthesis.

^a^ All values are from January 1 to June 30, 2015.

**Figure 1.**
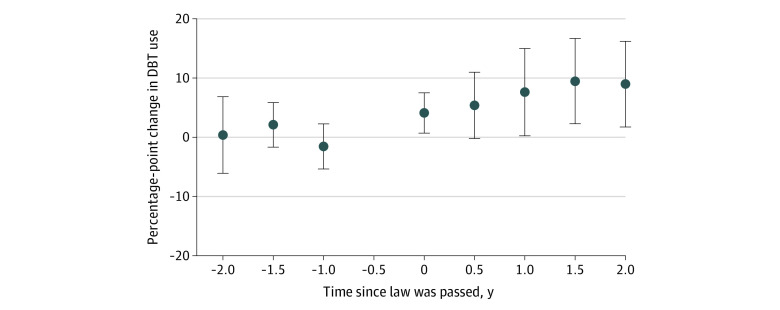
Change in Digital Breast Tomosynthesis (DBT) Use in States That Enacted a DBT Coverage Mandate vs States That Did Not No value is shown for the −0.5 period because this was the reference period. Error bars indicate 95% CIs.

In early 2015, among women living in states that eventually passed a DBT coverage mandate, 16% who underwent mammography were screened with DBT. Among women living in states that never passed a mandate, 11% were screened with DBT (Table). The event-study model suggested that DBT use increased faster in states with a coverage mandate than in those without one. Beginning 1 year after enactment of a coverage mandate, DBT use increased by 7.6 percentage points (95% CI, 0.3-15.0 percentage points) more in states with a mandate compared with nonmandate states. After 2 years, DBT use increased 9.0 percentage points (95% CI, 1.8-16.3 percentage points; *P* = .02) more in mandate states compared with nonmandate states (Figure 1 and eTable 3 in the Supplement). Findings from sensitivity analyses using a simplified difference-in-differences model were similar (eTables 9 and 10 in the Supplement).

**Figure 2.**
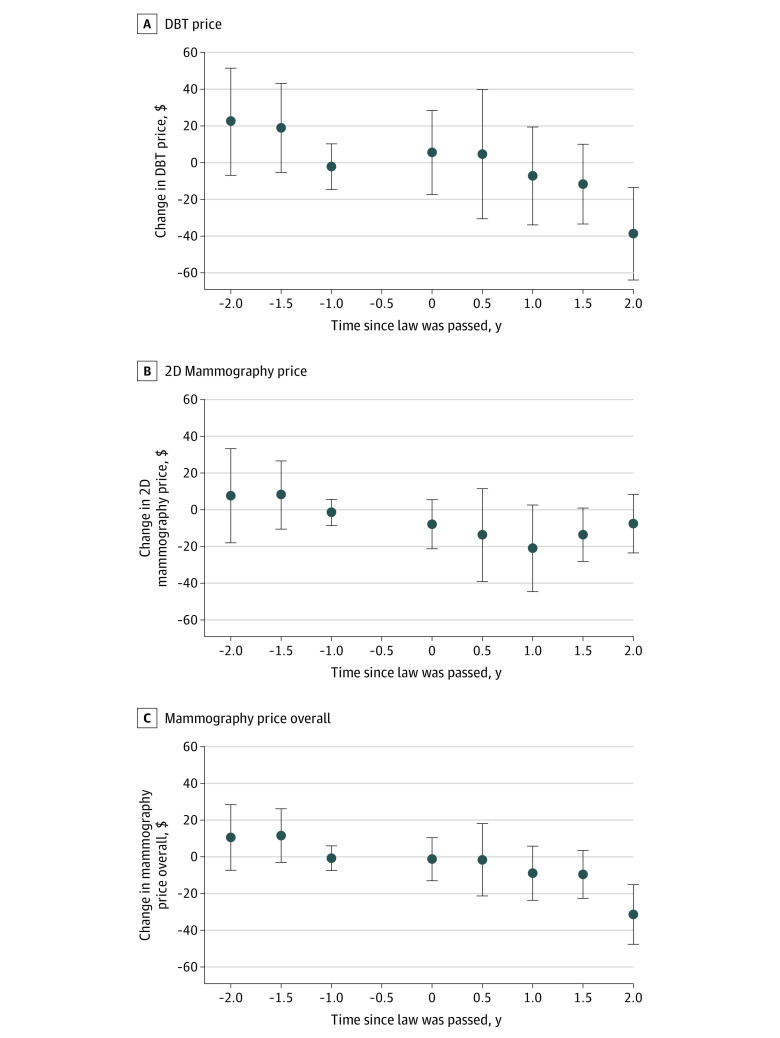
Changes in Digital Breast Tomosynthesis (DBT), 2-Dimensional (2D) Mammography, and Overall Mammography Prices in States That Enacted a DBT Coverage Mandate vs States That Did Not Prices were adjusted for inflation to 2019 dollars. No value is shown for the −0.5 period because this was the reference period. Error bars indicate 95% CIs.

In early 2015, the mean (SD) cost of DBT was $311.36 ($131.80) among mammograms performed in states that eventually passed a mandate and $347.05 ($149.43) in nonmandate states (Table). Using the event-study approach, 2 years after passage of a mandate, DBT price had decreased in mandate states compared with nonmandate states by $38.7 (95% CI, $13.4-$63.9; *P* = .003) (Figure 2, A and eTable 4 in the Supplement). Models accounting for 2D mammography price produced similar results (eFigures 2 and 3 in the Supplement). We did not observe a statistically significant change in the price of 2D mammography in mandate states compared with nonmandate states at 2 years (–$7.6; 95% CI, $–23.6 to $–8.4) (Figure 2, B and eTable 5 in the Supplement). However, the mean price of mammography overall (2D mammography and DBT) decreased in mandate states compared with nonmandate states at 2 years ($–31.4; 95% CI, $–15.2 to $–47.6), reflecting the decrease in price among women screened with DBT (Figure 2, C and eTable 6 in the Supplement). Findings from difference-in-differences models were similar (eTables 9 and 10 in the Supplement).

**Figure 3.**
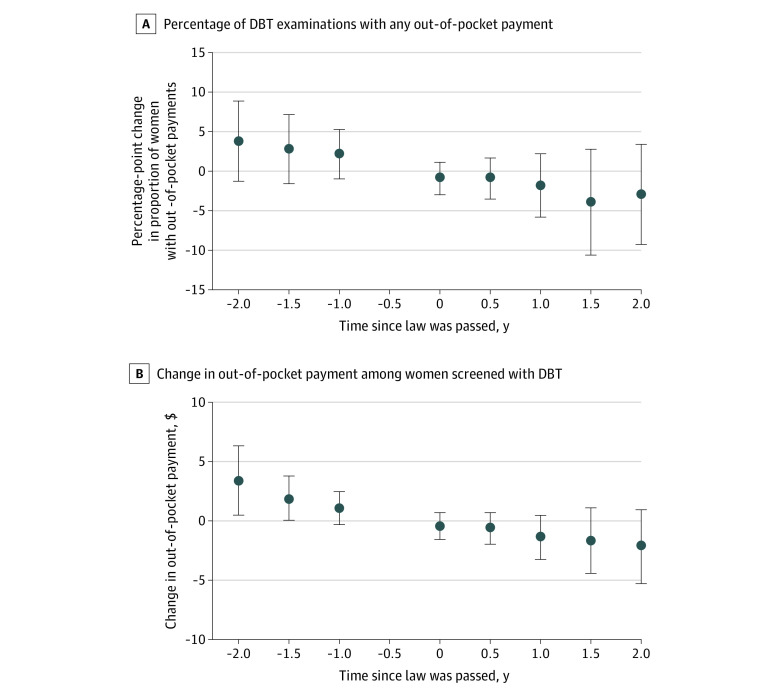
Changes in the Proportion of Women Screened With Digital Breast Tomosynthesis (DBT) With Any Out-of-Pocket Payment and in Out-of-Pocket Payments Among Women Screened With DBT in States That Enacted a DBT Coverage Mandate vs States That Did Not No value is shown for the −0.5 period because this was the reference period. Error bars indicate 95% CIs.

When we examined the association between state mandates and out-of-pocket payments among women screened with DBT, we found that at the start of the study period, few women had any out-of-pocket payments for DBT (7% each in mandate states and nonmandate states) (Table). We did not observe a statistically significant change in the proportion of women who had any out-of-pocket payments for DBT in mandate states compared with nonmandate states at 2 years after mandate enactment (–3.0%; 95% CI, –9.3% to 3.4%) (Figure 3, A and eTable 7 in the Supplement). At 2 years after mandate enactment, we also did not observe a significant change in mean out-of-pocket payments among women who were screened with DBT ($−2.1; 95% CI, $–5.3 to $1.0; *P* = .18) (Figure 3, B and eTable 8 in the Supplement).

## Discussion

In this study, we found that mandates requiring first-dollar insurance coverage for DBT were associated with a 9.0-percentage point increase in DBT use 2 years after law enactment. We also found that the price of DBT decreased in mandate states compared with nonmandate states by $38.7 per screening 2 years after mandate enactment. In addition, our findings demonstrated that out-of-pocket payments were rare and did not increase in nonmandate states compared with mandate states even as DBT use increased considerably.

A central policy objective of coverage mandates is to ensure access to a particular medical technology or service by protecting patients from financial liability. Our results suggest that women in states with coverage mandates were more likely to begin to use DBT for breast cancer screening. This finding is consistent with other studies^17,18,19^ that have found that expanding coverage and, in particular, eliminating cost sharing may be associated with increased use of specific health services. For example, several studies have demonstrated greater use of screening mammography among women with first-dollar coverage.^17,18,19^

However, our study also raises new questions about the mechanism by which mandates are associated with increased use of an emerging technology. We observed that the proportion of women screened with DBT who had any out-of-pocket payment was low even before passage of any coverage mandates. As DBT use increased in both mandate and nonmandate states, we did not observe an increase in the proportion of women with out-of-pocket payments in nonmandate states compared with mandate states. These finding suggest that out-of-pocket payments were rare regardless of whether a mandate was in place and likely were not the primary barrier to DBT use for most women.

Instead, our findings raise the possibility that DBT coverage mandates contributed to broader adoption of DBT among radiologists, perhaps by reducing uncertainty about payment. In states that had passed mandates, we observed both greater DBT use and a relative reduction in DBT price compared with nonmandate states. One possible explanation for these findings is that by ensuring payment, coverage mandates may have encouraged radiologists and health care institutions to enter the market and offer DBT. This scenario may have been associated with a lower relative price in at least 2 ways. First, when more radiologists offer DBT, insurers may have greater ability to negotiate lower prices, leading to lower prices or slower increases in prices overall. Second, early entrants may be radiologists or health care organizations that offer services at higher prices. If mandates incentivize new entrants to the market who tend to offer services at a lower price compared with established mammography services, the typical price in the market will decrease even though individual services prices do not change. However, other explanations for these findings are possible. For example, coverage mandates might be perceived as an endorsement of the technology by state officials, increasing interest in use of the new technology.

Our findings also suggest that coverage mandates can have complex implications for value in health care. Digital breast tomosynthesis has some advantages over 2D mammography, including lower recall rates.^5,20^ However, a recent cost-effectiveness study by Lowry et al^21^ found that DBT was not cost-effective at typical willingness-to-pay thresholds largely because the health gains from DBT were modest compared with those from 2D digital mammography, whereas the marginal costs were high.^21^ Coverage mandates, when put in place without regard to cost-effectiveness, have the potential to contribute to inefficiencies in health care if they encourage use of low-value services.

However, we also observed that DBT prices decreased in mandate states compared with states that had not passed a mandate. This lower price may be associated with improved cost-effectiveness. Lowry et al^21^ reported that a $30 reduction in the price of DBT may place it in the cost-effective range, a reduction in price similar to what we observed. However, whether coverage mandates truly improve efficiency remains uncertain and depends on whether the coverage mandate lowered the absolute price of DBT (rather than simply the change in DBT price in mandate states compared with states that had not passed a mandate) and whether there were concurrent changes in the price of 2D mammography. Whether mandates improve efficiency also depends on whether the association we observed reflected a causal relationship, but causation could not be assessed in this study.

Although this study focused on the specific case of DBT, insurance coverage mandates are commonly deployed policy strategies intended to improve patient access to a broad range of health services and to protect patients from financial liability. Of note, the Patient Protection and Affordable Care Act mandates coverage of many health services, including evidence-based preventive services. State legislatures have also issued coverage mandates for a diverse set of services including fertility treatment, assessment of and treatment for children with autism, cancer treatment, and diabetes medications.^22^ Such mandates have historically been controversial because they may be associated with improved access but also may contribute to the increasing cost of premiums.^14^ Our work adds another dimension to the understanding of the effects of coverage mandates. Although mandates may have the potential to contribute to health care costs by facilitating the use of more health services (including low-value services), they may also encourage competition in local markets, which may lead to lower per-unit prices.

### Limitations

This study has limitations. First, we used data from an affiliation of associated insurance plans, and results may not be applicable to other populations. Second, coverage mandates typically do not apply to self-insured firms, Medicare, or Medicaid. Thus, effects in other insurance markets may be variable. There are also important limitations to using claims data. Although we used a validated algorithm to identify screening mammography, claims may be subject to error or misclassification. Our approach also focused on the price of the initial test rather than the screening episode (which would include follow-up testing). Thus, we did not capture downstream changes in the use or price of these subsequent tests. In addition, this was an observational study. Although we used a study design intended to limit confounding, unmeasured confounding is possible and may explain some of our findings. For example, other concurrent legislative policies (such as breast density notification legislation) or concurrent coverage decisions from insurers (such as changes in Blue Cross Blue Shield policies to allow use of DBT even absent a mandate) may contribute to confounding.^23^ Similarly, states that enacted coverage mandates may have been more eager to adopt DBT even before legislation or may have had fundamentally different trajectories in DBT costs. Although our event-study plots did not show statistically significant differences in DBT use or price before legislation enactment, it is important to acknowledge that preperiod trends in DBT use or cost in mandate states may have influenced our results.

## Conclusions

In the present study, state-level mandates requiring coverage for screening DBT were associated with meaningful increases in DBT use and relative reductions in DBT price. Our findings overall suggest that mandates may encourage DBT use but also may have more complex implications for the associations among technology adoption, price, and value.

## Notes

Supplement.
eMethods.
**eTable 1.** Algorithm for Identifying Screening Mammograms **eTable 2.** Details of State-Level Coverage Mandates **eTable 3.** DBT Use (Percentage Point Change) **eTable 4.** DBT Cost **eTable 5.** 2D Cost **eTable 6.** Overall Screening Mammogram Cost (DBT or 2D) **eTable 7.** Percent of Women Screened With DBT With Any Out-of-Pocket Payment **eTable 8.** Event Study Model of Out-of-Pocket Payment Among Women Screened With DBT ($) **eTable 9.** Difference-in-Differences Estimates **eTable 10.** Difference-in-Differences Estimates Excluding Transition Period **eFigure 1.** Timing of State Insurance Coverage Mandate Legislation **eFigure 2.** Mean DBT Price, Adjusted for 2D Price **eFigure 3.** Mean DBT Price, Less Mean 2D Price
