# Supplementary material for: Insurance Coverage Mandates and the Adoption of Digital Breast Tomosynthesis
Source: JAMA Netw Open. 2022 Mar 25;5(3):e224208. doi: 10.1001/jamanetworkopen.2022.4208 (PMC8956980; doi:10.1001/jamanetworkopen.2022.4208)
Supplement: Supplement. — eMethods. eTable 1. Algorithm for Identifying Screening Mammograms eTable 2. Details of State-Level Coverage Mandates eTable 3. DBT Use (Percentage Point Change) eTable 4. DBT Cost eTable 5. 2D Cost eTable 6. Overall Screening Mammogram Cost (DBT or 2D) eTable 7. Percent of Women Screened With DBT With Any Out-of-Pocket Payment eTable 8. Event Study Model of Out-of-Pocket Payment Among Women Screened With DBT ($) eTable 9. Difference-in-Differences Estimates eTable 10. Difference-in-Differences Estimates Excluding Transition Period eFigure 1. Timing of State Insurance Coverage Mandate Legislation eFigure 2. Mean DBT Price, Adjusted for 2D Price eFigure 3. Mean DBT Price, Less Mean 2D Price [file jamanetwopen-e224208-s001.pdf]

Supplementary Online Content

Richman IB, Long JB, Kyanko KA, Xu X, Gross CP, Busch SH. Insurance coverage mandates and the adoption of digital breast tomosynthesis. *JAMA Netw Open*. 2022;5(3):e224208. doi:10.1001/jamanetworkopen.2022.4208

eMethods.
eTable 1. Algorithm for Identifying Screening Mammograms
eTable 2. Details of State-Level Coverage Mandates
eTable 3. DBT Use (Percentage Point Change)
eTable 4. DBT Cost
eTable 5. 2D Cost
eTable 6. Overall Screening Mammogram Cost (DBT or 2D)
eTable 7. Percent of Women Screened With DBT With Any Out-of-Pocket Payment
eTable 8. Event Study Model of Out-of-Pocket Payment Among Women Screened With DBT ($)
eTable 9. Difference-in-Differences Estimates
eTable 10. Difference-in-Differences Estimates Excluding Transition Period
eFigure 1. Timing of State Insurance Coverage Mandate Legislation
eFigure 2. Mean DBT Price, Adjusted for 2D Price
eFigure 3. Mean DBT Price, Less Mean 2D Price

This supplementary material has been provided by the authors to give readers additional information about their work.

© 2022 Richman IB et al. *JAMA Network Open*.

eMethods.

Identifying screening mammograms:

To identify screening mammograms, we adapted a validated algorithm that distinguishes diagnostic and screening mammograms. From among all mammograms, the algorithm first excludes claims for a mammogram with a Healthcare Common Procedure Coding System (HCPCS) code indicating a diagnostic mammogram. Then, the algorithm excludes mammograms performed within 9 months of a previous mammogram. Lastly, the algorithm excludes mammograms performed within 365 days of a breast cancer diagnosis, defined by ICD 9 or 10 codes. We used a 9-month lookback period to be able to maximize the included mammograms. Although this may reduce the specificity of the algorithm, it should not differentially affect mammograms done in different states.

Sensitivity Analyses and Alternate Specifications:

Evaluating Changes in DBT Price, adjusting for 2D price

In addition to the outcomes detailed in our main manuscript, we also modeled the relationship between coverage mandates and DBT price but included 2D price as a covariate. This model accounts for changes in 2D price which may impact DBT price, since DBT is billed as an add-on to 2D, but are unrelated to DBT coverage mandates. This model may also help account for differential pre-mandate trends observed in both the price of 2D mammography and DBT. This alternate specification is presented in eFigure 2 and eTable 3b.

As an alternative to this specification, we also modeled the difference between mean DBT price and mean 2D price as the outcome (i.e. rather than mean DBT price adjusted for 2D price). Similar to adjusting for 2D price, this outcome captures changes in DBT price after accounting for change in 2D prices that would not be affected by coverage mandates. To calculate this, we took the difference between the mean DBT price and mean 2D price for each state at each time point. Using this value as the outcome, we fit an event study model identical to that used in our main analyses. These alternate specification is presented in eFigure 3.

2X2 Difference-in Differences:

As an alternative to an event-study design, we also performed a traditional 2x2 difference-in-differences analysis with a single before/after time period. Here, we considered the four states with at least 2 years of follow up time (CT, IL, PA, NY) as mandate states and all non-mandate states as control states. States with <2 years of follow up post mandate (n=12) and the state of Indiana were excluded. The pre-mandate period spanned from 1/1/2016-12/31/2016 and post mandate was from 1/1/2017 to 6/30/2019. We also performed a second analysis using the same approach but excluding the period from 1/1/2016-1/1/2017, as some states had already enacted legislation during this transition period. Our analyses used a simple specification (below). We clustered standard errors by state to account for repeated measures on each state.

$$Y_{outcome} = \beta_{period} + \beta_{intervention} + \beta_{period*intervention} + \varepsilon$$

© 2022 Richman IB et al. *JAMA Network Open*.

eTable 1. Algorithm for identifying screening mammograms

Description	Codes (CPT/HCPCS unless otherwise noted)
Screening mammography	77057, 77067, G0202, +GG (+77063 DBT)
Diagnostic mammography	G0204, G0206, 77055, 77056, 77065, 77066, +77051 (add-on); DBT indicated by 77061, 77062 as part of main procedure or +G0279 (add-on)
History of breast cancer	ICD-9 Diagnosis Codes: 174.*, 233.0, V10.3<br>ICD-10 Diagnosis Code: C50.*; D05.*, Z85.3*, Z86.000

© 2022 Richman IB et al. *JAMA Network Open*.

eTable 2. Details of State-Level Coverage Mandates

State	Date of Insurance Coverage Mandate	Description of legislation	Cost Sharing prohibited with coverage mandate
Arkansas (AR)	8/3/2017	Requires insurers to cover cost of 3D mammograms and ultrasounds for routine screenings and policy shall not impose copayment or deductible	Yes
Colorado (CO)	1/1/2021	Mandates coverage for breast cancer screening with noninvasive imaging	Yes
Connecticut (CT)	1/1/2017	Requires coverage for tomosynthesis, in addition to a mammogram, and to allow women the option to choose either process	Cost sharing eliminated 1/1/2018
District of Columbia (DC)	3/22/2019	Requires insurers to cover a baseline and annual mammogram for women, including a 3D mammogram	Yes
Illinois (IL)	7/1/2016	Provides that if coverage includes mammogram, the coverage shall not impose a deductible, coinsurance, copayment, or any other cost-sharing requirement.	Yes
Indiana (IN)	7/1/2013	Health insurance must provide coverage for appropriate medical screening for females at least 40 years of age who have been found to have high breast density	N/A
Kentucky (KY)	7/3/2017	Requires insurers to cover cost of mammograms and screenings and policy shall not impose copayment or deductible	Yes
Louisiana (LA)	1/1/2019	Requires insurance coverage of DBT by adding DBT to current definition of minimum mammography examination	Yes
Maryland (MD)	1/1/2018	Expands health insurance for coverage of breast cancer screenings to include coverage for digital tomosynthesis.	Yes
Minnesota (MN)	1/1/2020	Requires coverage for routine screening procedures, including	Yes

© 2022 Richman IB et al. *JAMA Network Open*.

		mammograms and digital tomosynthesis	
Missouri (MO)	8/1/2018	Insurers required to cover cost of 3D mammography	Yes
New Jersey (NJ)	8/1/2018	Insurance plans must cover digital tomosynthesis to detect or screen for breast cancer	Yes
New Hampshire	8/7/2018	Expands definition of mammography to include 3D	Cost sharing allowed after 9/10/2019
New York (NY)	1/1/2017	Mandates medically necessary coverage for DBT screenings without copay or deductibles	Yes
Oklahoma (OK)	11/1/2018	Added DBT to definition of low-dose mammography and requires insures to include coverage for DBT	Yes
Pennsylvania (PA)	10/1/2015	3D mammograms must be covered at no cost to women	Yes
Texas (TX)	9/1/2017	Requires insurers to cover 3D mammograms	Yes
Vermont (VT)	1/1/2019	Requires insurers to cover breast imaging services without imposing cost-sharing requirements	Yes
Washington (WA)	6/7/2018	Insurers must include coverage for DBT or 3D mammography under the same terms and conditions currently allowed for mammography, so deductibles and cost sharing is prohibited	Yes

© 2022 Richman IB et al. *JAMA Network Open*.

eTable 3. DBT Use (Percentage point change)

Period	Estimate*	95% CI	Adjusted for ASO membership**	95% CI
-2	0.4	-6.0-6.9	1.1	-7.2-4.5
-1.5	2.1	-1.6-5.9	1.2	-2.2-4.6
-1	-1.5	-5.3-2.3	-1.7	-5.5-2.1
0	4.1	0.7-7.5	4.1	0.5-7.7
0.5	5.4	-0.2-11.0	5.3	-0.2-1.1
1	7.6	0.3-15.0	7.0	-0.4-1.4
1.5	9.4	2.3-16.7	8.7	0.2-1.6
2	9.0	1.8-16.3	8.1	0.7-15.5
*Estimates from primary event-study specification<br>**Estimates from model adjusting for % population with Administrative Services Only (ASO) plans				

eTable 4. DBT Cost

Period	Estimate*	95% CI	Adjusted for ASO membership**	95% CI	Adjusted for 2D price***	95% CI
-2	22.3	-6.9-51.4	21.5	-9.8-52.9	15.7	-17.5-48.8
-1.5	18.9	-5.3-43.1	18.4	-6.6-43.5	11.8	-7.5-31.0
-1	-2.2	-14.6-10.2	-2.3	-14.9-10.3	-1.0	-12.2-10.2
0	5.5	-17.4-28.4	5.5	-17.5-28.4	12.3	-6.8-31.5
0.5	4.6	-30.6-39.8	4.5	-30.6-39.7	16.6	-9.3-42.5
1	-7.3	-33.9-19.4	-7.6	-34.2-19.0	11.0	-10.4-32.4
1.5	-11.9	-33.8-10.1	-12.2	34.1-9.7	-0.01	-21.2-21.3
2	-38.7	-63.9- -13.4	-39.1	-64.2- -14.0	-32.1	-53.3- -10.9
*Estimates from primary event-study specification<br>**Estimates from model adjusted for % population with Administrative Services Only (ASO) plans<br>***Estimates from model adjusted for 2D cost						

© 2022 Richman IB et al. *JAMA Network Open*.

eTable 5. 2D Cost

Period	Estimate*	95% CI	Adjusted for ASO membership**	95% CI
-2	7.6	-18.0-33.2	6.5	-19.5-32.6
-1.5	8.2	-10.6-26.9	7.5	-11.7-26.8
-1	-1.4	-8.5-5.8	-1.5	-8.7-26.8
0	-7.9	-21.3-5.5	-7.9	-21.2-5.5
0.5	-13.8	-39.1-11.5	-13.9	-39.1-11.2
1	-21.0	-44.6-2.6	-21.5	-44.8-1.9
1.5	-13.6	-28.2-0.9	-14.1	-28.6-0.3
2	-7.6	-23.6-8.4	-8.2	-24.0-7.5

*Estimates from primary event-study specification
**Estimates from model adjusted for % population with Administrative Services Only (ASO) plans

eTable 6. Overall Screening Mammogram Cost (DBT or 2D)

Period	Estimate*	95% CI	Adjusted for ASO membership**	95% CI
-2	10.5	-7.4-28.4	7.7	-10.5-25.9
-1.5	11.5	-3.1-26.1	9.8	-5.5-25.2
-1	-0.8	-7.5-5.9	-1.1	-8.0-5.7
0	-1.2	-12.8-10.4	-1.2	-12.9-10.5
0.5	-1.7	-21.3-18.0	-1.9	-21.5-17.7
1	-8.9	-23.6-5.8	-10.1	-25.0-4.7
1.5	-9.6	-22.6-3.5	-11.0	-24.1-2.1
2	-31.4	-47.6- -15.2	-33.0	-49.5- -16.6

*Estimates from primary event-study specification
**Estimates from model adjusted for % population with Administrative Services Only (ASO) plans

eTable 7. Percent of women screened with DBT with any out-of-pocket payment

Period	Estimate*	95% CI	Adjusted for ASO membership**	95% CI
-2	3.8	-1.3-8.9	5.2	-1.6-12.1
-1.5	2.7	-1.7-7.2	3.6	-1.6-8.9
-1	2.2	-1.0-5.3	2.4	-1.1-5.8
0	-0.9	-3.0-1.1	-1.0	-3.2-12.9
0.5	-0.9	-3.5-1.7	-0.8	-3.5-1.9
1	-1.8	-5.9-2.3	-1.2	-4.4-2.1
1.5	-3.9	-10.6-2.9	-3.1	-9.1-2.8
2	-3.0	-9.3-3.4	-2.0	-7.5-3.3

*Estimates from primary event-study specification
**Estimates from model adjusted for % population with Administrative Services Only (ASO) plans

© 2022 Richman IB et al. *JAMA Network Open.*

eTable 8. Event study model of out-of-pocket payment among women screened with DBT ($)

Period	Estimate*	95% CI	Adjusted for ASO Membership**	95% CI
-2	3.4	0.5-6.3	3.7	-0.2-7.3
-1.5	1.9	0.0-3.7	2.1	-0.1-4.3
-1	1.1	-0.3-2.4	1.1	-0.3-2.6
0	-0.4	-1.6-0.7	-0.4	-1.6-0.7
0.5	-0.6	-2.0-0.8	-0.6	-1.9-0.8
1	-1.4	-3.2-0.5	-1.2	-2.8-0.4
1.5	-1.7	-4.4-1.0	-1.5	-3.9-0.9
2	-2.1	-5.3-1.0	-1.9	-4.7-0.8

*Estimates from primary event-study specification
**Estimates from model adjusted for % population with Administrative Services Only (ASO) plans

© 2022 Richman IB et al. *JAMA Network Open*.

eTable 9. Difference-in-Differences Estimates

	Pre period		Post Period			
**Outcome (95% CI)**	**No mandate**	**Mandate****	**No mandate**	**Mandate****	**Difference in Differences**	**p**
DBT Use, %	17.3 (10.2-24.3)	27.2 (24.3-30.0)	48.0 (39.3-56.7)	66.6 (62.7-70.5)	8.7 (3.8-13.7)	0.001
Mean DBT price	295.3 (246.2-344.4)	308.6 (297.0-320.3)	329.0 (283.7-374.2)	309.6 (295.4-323.9)	-32.6 (-52.1- -13.2)	0.002
Mean 2D price	252.5 (214.3-290.7)	276.8 (271.2-282.3)	247.3 (217.3-277.4)	265.5 (262.7-268.2)	-6.1 (-18.6-6.3)	0.32
Mean price of any mammo	261.6 (220.7-302.5)	288.2 (283.5-292.9)	289.6 (250.1-329.1)	293.5 (285.7-301.3)	-22.7 (-37.2- -8.1)	0.003
Mean OOP* cost for DBT	4.7 (2.8-6.6)	2.4 (-0.7-5.4)	6.2 (2.5-9.9)	1.1 (0.5-1.7)	-2.8 (-6.3-0.7)	0.12
Mean OOP cost if any	66.2 (53.1-79.2)	48.8 (45.1-52.5)	77.9 (63.7-92.1)	69.6 (63.3-76.0)	9.1 (-3.6-21.8)	0.15

*OOP=Out of pocket
** Note that only 4 mandate states (CT, NY, IL, PA) are included in this analysis

© 2022 Richman IB et al. *JAMA Network Open.*

eTable 10. Difference-in-Differences Estimates Excluding Transition Period**

	**Pre period**		**Post Period**			
**Outcome (95% CI)**	**No mandate**	**Mandate*** **	**No mandate**	**Mandate*** **	**Difference in Differences**	**p**
DBT Use, %	12.7<br>6.5-18.8	17.0<br>(16.5-17.4)	48.0<br>(39.2-56.8)	66.6<br>(62.7-70.5)	14.3<br>(7.6-21.0)	<0.001
Mean DBT price	289.8<br>(240.7-348.9)	281.9<br>(263.3-300.6)	329.0<br>(283.7-374.2)	309.6<br>(295.4-323.9)	-11.4<br>(-32.7- 9.9)	0.27
Mean 2D price	253.5<br>(214.0-293.0)	276.8<br>(271.2-282.3)	247.3<br>(217.2-277.4)	265.5<br>(262.7-268.2)	-6.0<br>(-19.8-7.8)	0.38
Mean price of any mammo	259.7<br>(217.6-301.7)	279.1<br>(274.9-283.2)	289.6<br>(250.1-329.1)	293.5<br>(285.7-301.3)	-15.5<br>(-28.2- -2.3)	0.02
Mean OOP cost for DBT	4.7<br>(3.3-6.4)	2.7<br>(-0.7-5.4)	6.2<br>(2.5-9.9)	1.1<br>(0.5-1.7)	-3.0<br>(-6.7-0.7)	0.11
Mean OOP cost if any	63.7<br>(52.1-75.4)	39.2<br>(34.1-44.4)	77.9<br>(63.7-92.1)	69.6<br>(63.3-76.0)	16.2<br>(5.0-27.5)	0.006

*OOP=Out of pocket
**Estimates exclude the period from 1/1/2016-1/1/2017
*** Note that only 4 mandate states (CT, NY, IL, PA) are included in this analysis

© 2022 Richman IB et al. *JAMA Network Open.*

eFigure 1. Timing of State Insurance Coverage Mandate Legislation

State	Time 0 Reference	2015	2015.5	2016	2016.5	2017	2017.5	2018	2018.5	2019
**Law States**										
PA	1/1/16									
IL	7/1/16									
CT	1/1/17									
NY	1/1/17									
KY	7/1/17									
TX	1/1/18									
MD	1/1/18									
AR	1/1/18									
WA	7/1/18									
LA	1/1/19									
NH	1/1/19									
NJ	1/1/19									
VT	1/1/19									
MO	1/1/19									
OK	1/1/19									

eFigure 1: The figure indicates the timing of enactment of mandate legislation. We considered “time zero” to be the first period in which a legislative mandate was fully enacted. One exception was Kentucky, which enacted a mandate on 7/3/2017, so we considered the period beginning 7/1/2017 to be time zero, rather than the period beginning 1/1/2018.

© 2022 Richman IB et al. *JAMA Network Open*.

eFigure 2. Mean DBT Price, Adjusted for 2D Price

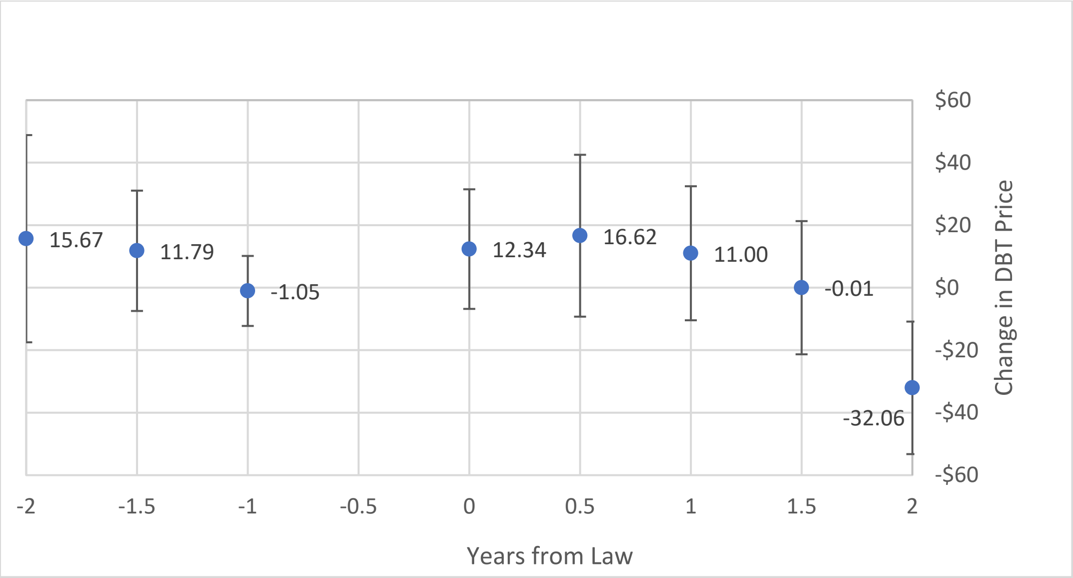

eFigure 2~~a~~: Figure depicts the change in DBT prices in states that passed a DBT coverage mandate relative to states that did not pass a mandate over the study period, adjusted for 2D price. Error bars indicate 95% confidence intervals. Prices are adjusted for inflation to 2019 dollars. No value is shown for the “-0.5” period as this is the reference period.

© 2022 Richman IB et al. *JAMA Network Open*.

eFigure 3. Mean DBT Price, Less Mean 2D Price

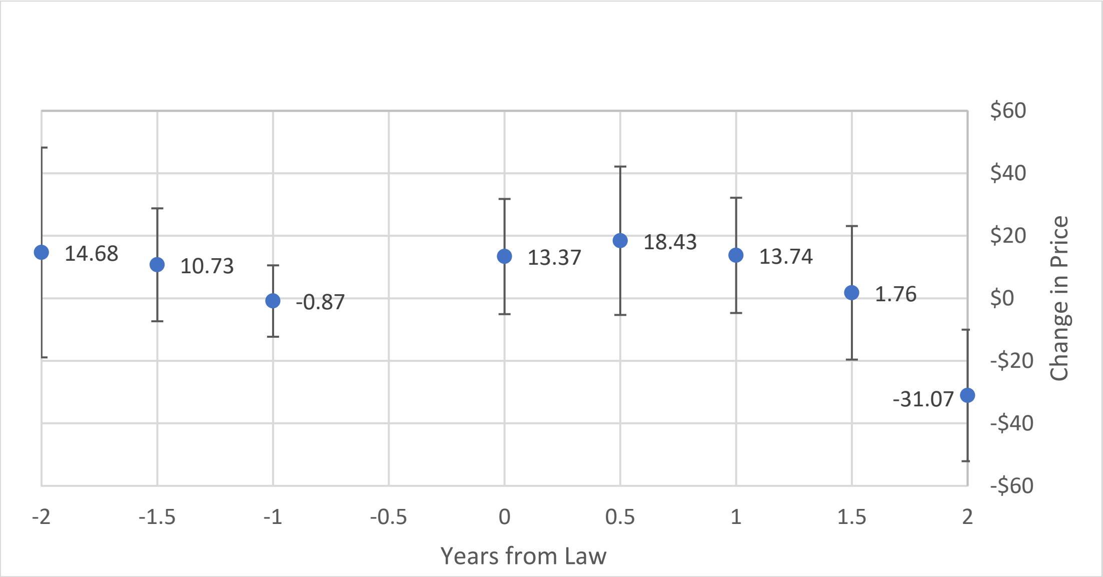

eFigure 32b: Figure depicts the difference in DBT price less 2D price in states that passed a DBT coverage mandate relative to states that did not pass a mandate over the study period. Error bars indicate 95% confidence intervals. Prices are adjusted for inflation to 2019 dollars. No value is shown for the “-0.5” period as this is the reference period.

© 2022 Richman IB et al. *JAMA Network Open.*
